# Supplementary material for: Parvovirus B19 Outbreak in Israel: Retrospective Molecular Analysis from 2010 to 2023
Source: Viruses. 2024 Mar 20;16(3):480. doi: 10.3390/v16030480 (PMC10974090; doi:10.3390/v16030480)
Supplement: Supplementary file 1 [file viruses-16-00480-s001.zip › viruses-2896045-supplementary.pdf]

Supplementary Material

Table S1: Data describing sequenced samples from Israel.
NA indicates non-available information.

Sequence number	Date	Year	Age Group	Gender	City	Country
274393	2012-12-31	2012	11-20	male	Kfar Saba	Israel
390408	2012-08-22	2012	31-40	female	Zfat	Israel
284066	2012-11-04	2012	61-70	female	Ashdod	Israel
286565	2013-05-19	2013	41-50	female	Ramat Gan	Israel
289912	2013-07-02	2013	51-60	male	Kadima Tzoran	Israel
305713	2014-01-15	2014	41-50	female	Tel Aviv	Israel
314918	2014-04-13	2014	11-20	male	NA	Israel
318400	2014-08-06	2014	51-60	female	Ramat Gan	Israel
320189	2014-07-03	2014	NA	male	NA	Israel
339097	2015-01-18	2015	21-30	female	Aatzem	Israel
352434	2015-07-08	2015	41-50	male	Rosh Haayin	Israel
366548	2015-12-20	2015	21-30	female	Modiin	Israel
391900	2016-08-24	2016	51-60	female	Tel Aviv	Israel
389539	2016-08-04	2016	41-50	female	Holon	Israel
408686	2017-01-22	2017	31-40	female	Ashkelon	Israel
411433	2017-02-13	2017	11-20	female	Rishon Letzion	Israel
411864	2017-02-16	2017	NA	NA	Rishon Letzion	Israel
414188	2017-03-09	2017	0-10	male	Rishon Letzion	Israel
449145	2018-01-17	2018	0-10	NA	Maale Levona	Israel
452009	2018-02-06	2018	11-20	male	Ramat Gan	Israel
458100	2018-04-01	2018	41-50	female	Beer Sheva	Israel
495975	2019-01-14	2019	NA	NA	Shfaram	Israel
496304	2019-01-15	2019	21-30	female	Masada	Israel
509602	2019-04-14	2019	31-40	female	Iksal	Israel
517902	2019-06-19	2019	41-50	male	Tel Aviv	Israel
551188	2020-03-01	2020	41-50	female	Mazkeret Batya	Israel
551061	2020-03-01	2020	31-40	female	Lod	Israel
559102	2020-04-06	2020	11-20	female	Kfar Bara	Israel
659962	2021-08-19	2021	21-30	female	Patish	Israel
634558	2021-01-04	2021	0-10	male	Masada	Israel
638908	2021-02-03	2021	51-60	male	Lakia	Israel
642423	2021-03-10	2021	11-20	female	Mashad	Israel
739565	2023-03-30	2023	11-20	male	Tel Aviv	Israel
743132	2023-04-27	2023	0-10	male	Lod	Israel
744582	2023-05-03	2023	31-40	female	Kfar Habad	Israel

745152	2023-05-01	2023	21-30	female	Petach Tikva	Israel
745241	2023-05-01	2023	11-20	female	Ashdod	Israel
728	2023-05-29	2023	0-10	female	NA	Israel
454053	2018-02-19	2018	0-10	NA	Jaljulia	Israel
391966	2016-08-25	2016	51-60	female	Cohav Yair	Israel
712072	2022-10-06	2022	11-20	female	Modiin	Israel
731921	2013-01-02	2013	0-10	female	Givat Shmuel	Israel
553925	2020-03-09	2020	11-20	male	Kfar Bara	Israel

Table S2: raw sequence data
>638908
aaactgggcaataaactacacttttgatttccctggaattaatgcagatgccctccaccc
agacctccaaaccaccccaattgtcacagacaccagtatcagcagcagtggtggtgaaag
ctctgaagaactcagtgaaagcagctttttttaacctcatcaccccaggcgcctggaacac
tgaaaccccgcgctctagtacgcccatccccgggaccagttcaggagaatcatttgtcgg
aagcccagtttcctccgaagttgtagctgcatcgtgggaagaagccttctacacaccttt
agcagaccagtttcgtgaactgttagttggggttgattatgtgtgggacggtgtaagggg
tttacctgtgtgttgtgtacaacatattaacaatagtgggggaggcttgggactttgtcc
ccattgcattaatgtaggggcttggtataatggatggaaatttcgagaatttaccccaga
tttggtgcggtgtagctgccatgtgggagcttctaatcccttttctgtgctaacctgcaa
aaaatgtgcttacctgtctggattgcaaagctttgtagattatgagtaaagaaagtggca
aatggtgggaaagtgatgataaatttgctaaagctgtgtatcagcaatttgtggaatttt
atgaaaaggttactggaacagacttagagcttattcaaatattaaaagatcactataata
tttctttagataatcccctagaaaacccatcctctttgtttgacttagttgctcgtatta
aaaataaccttaaaaactctccagacttatatagtcatcattttcaaagtcatggacagt
tatctgaccacccccatgccttatcatccagtagcagtcatgcagaacctagaggagaaa
atgcagtattatctagtgaagacttacacaagcctgggcaagttagcgtacaactacccg
gtactaactatgttgggcctggcaatgagctacaagctgggcccccgcaaagtgctgttg
acagtgctgcaaggattcatgactttaggtatagccaactggctaagttgggaataaatc
catatactcattggacggtagcagatgaaga
>274393
aaactgggcaataaactacacttttgatttccctggaattaatgcagatgccctccaccc
agacctccaaaccaccccaattgtcacagacaccagtatcagcagcagtggtggtgaaag
ctctgaagaactcagtgaaagcagctttttttaacctcatcaccccaggcgcctggaacac
tgaaaccccgcgctctagtacgcccatccccgggaccagttcaggagaatcatttgtcgg
aagcccagtttcctccgaagttgtagctgcatcgtgggaagaagccttctacacaccttt
ggcagaccagtttcgtgaactgttagttggggttgattatgtgtgggacggtgtaagggg
tttacctgtgtgttgtgtgcagcatattaacaatagtgggggaggcttgggactttgtcc
ccattgcattaatgtaggggcttggtataatggatggaaatttcgagaatttaccccaga
tttggtgcggtgtagctgccatgtgggagcttctaatcccttttctgtgctaacctgcaa
aaaatgtgcttacctgtctggattgcaaagctttgtagattatgagtaaagaaagtggca
aatggtgggaaagtgatgataaatttgctaaagctgtgtatcagcaatttgtggaatttt
atgaaaagcttactggaacagacttagagcttattcaaatattaaaagatcattacaata
tttctttagataatcccctagaaaacccatcctctctgtttgacttagttgctcgtatta
aaaataaccttaaaaactctccagacttatatagtcatcattttcaaagtcatggacagt

tatctgaccacccccatgccttatcatccagtaacagtcatgcagaacctagaggagaaa
atgcagtattatctagtgaagacttacacaagcctgggcaagttagcgtacaactacccg
gtactaactatgttgggcctggcaatgagctacaagctgggcccccgcaaagtgctgttg
acagtgctgcaaggattcatgactttaggtatagccaactggctaagttgggaataaatc
catatactcattggacggtagcagatgaaga
>391966
aaactgggcaataaactacacttttgatttccctggaattaatgcagatgccctccaccc
agacctccaaaccaccccaattgtcacagacaccagtatcagcagcagtggtggtgaaag
ctctgaagaactcagtgaaagcagctttttttaacctcatcaccccaggcgcctggaacac
tgaaaccccgcgctctagtacgcccatccccgggaccagttcaggagaatcatttgtcgg
aagcccagtttcctccgaagttgtagctgcatcgtgggaagaagccttctacacaccttt
ggcagaccagtttcgtgaactgttagttggggttgattatgtgtgggacggtgtaagggg
tttacctgtgtgttgtgtgcagcatattaacaatagtgggggaggcttgggactttgtcc
ccattgcattaatgtaggggcttggtataatggatggaaatttcgagaatttaccccaga
tttggtgcggtgtagctgccatgtgggagcttctaatcccttttctgtgctaacctgcaa
aaaatgtgcttacctgtctgggttgcaaagctttgtagattatgagtaaagaaagtggca
aatggtgggaaagtgatgataaatttgctaaagctgtgtatcagcaatttgtggaatttt
atgaaaagcttactggaacagacttagagcttattcaaatattaaaagatcattacaata
tttctttagataatcccctagaaaacccatcctctctgtttgacttagttgctcgtatta
aaaataaccttaaaaactctccagacttatatagtcatcattttcaaagtcatggacagt
tatctgaccacccccatgccttatcatccagtaacagtcatgcagaacctagaggagaaa
atgcagtattatctagtgaagacttacacaagcctgggcaagttagcgtacaactacccg
gtactaactatgttgggcctggcaatgagctacaagctgggcccccgcaaagtgctgttg
acagtgctgcaaggattcatgactttaggtatagccaactggctaagttgggaataaatc
catatactcattggactgtagcagatgaaga
>659962
aaactgggcaataaactacacttttgatttccctggaattaatgcagatgccctccaccc
agacctccaaaccactccaattgtcacagacaccagtatcagcagcagtggtggtgaaag
ctctgaagaactcagtgaaagcagctttttttaacctcatcaccccaggcgcctggaacac
tgaaaccccgcgctctagtacgcccatccccgggaccagttcaggagaatcatttgtcgg
aagcccagtttcctccgaagttgtagctgcatcgtgggaagaagccttctacacaccttt
ggcagaccagtttcgtgaactgttagttggggttgattatgtgtgggacggtgtaagggg
tttacctgtgtgttgtgtgcagcatattaacaatagtgggggaggcttgggactttgtcc
ccattgcattaatgtaggggcttggtataatggatggaaatttcgagaatttaccccaga
tttggtgcggtgtagctgccatgtgggagcttctaatcccttttctgtgctaacctgcaa
aaaatgtgcttacctgtctgggttgcaaagctttgtagattatgagtaaagaaagtgaca
aatggtgggaaagtgatgataaatttgctaaagctgtgtatcagcaatttgtggaatttt
atgaaaagcttactggaacagacttagagcttattcaaatattaaaagatcattacaata
tttctttagataatcccctagaaaacccatcctctctgtttgacttagttgctcgtatta
aaaataaccttaaaaactctccagacttatatagtcatcattttcaaagtcatggacagt
tatctgaccacccccatgccttatcatccagtaacagtcatgcagaacctagaggagaaa
atgcagtattatctagtgaagacttacacaagcctgggcaagttagcgtacaactacccg
gtactaactatgttgggcctggcaatgagctacaagctgggcccccgcaaagtgctgttg
acagtgctgcaaggattcatgactttaggtatagccaactggctaagttgggaataaatc
catatactcattggactgtagcagatgaaga
>408686
aaactgggcaataaactacacttttgatttccctggaattaatgcagatgccctccaccc

agacctccaaaccaccccaattgtcacagacaccagtatcagcagcagtggtggtgaaag
ctctgaagaactcagtgaaagcagctttttttaacctcatcaccccaggcgcctggaacac
tgaaaccccgcgctctagtacgcccatccccgggaccagttcaggagaatcatttgtcgg
aagcccagtttcctccgaagttgtagctgcatcgtgggaagaagccttctacacaccttt
ggcagaccagtttcgtgaactgttagttggggttgattatgtgtgggacggtgtaagggg
tttacctgtgtgttgtgtgcagcatattaacaatagtgggggaggcctgggactttgtcc
ccattgcattaatgtaggggcttggtataatggatggaaatttcgagaatttaccccaga
tttggtgcggtgtagctgccatgtgggagcttctaatcccttttctgtgctaacctgcaa
aaaatgtgcttacctgtctggattgcaaagctttgtagattatgagtaaaaaaagtggca
aatggtgggaaagtgatgataaatttgctaaagctgtgtatcagcaatttgtggaatttt
atgaaaagcttactggaacagacttagagcttattcaaatattaaaagatcattacaata
tttctttagataatcccctagaaaacccatcctctctgtttgacttagttgctcgtatta
aaaataaccttaaaaactctccagacttatatagtcatcattttcaaagtcatggacagt
tatctgaccacccccatgccttatcatccagtaacagtcatgcagaacctagaggagaaa
atgcagtattatctagtgaagacttacacaagcctgggcaagttagcgtacaactacccg
gtactaactatgttgggcctggcaatgagctacaagctgggcccccgcaaagtgctgttg
acagtgctgcaaggattcatgactttaggtatagccaactggctaagttgggaataaatc
catatactcattggactgtagcagatgaaga
>744582
aaactgggcaataaactacacttttgatttccctggaattaatgcagatgccctccaccc
agacctccaaaccaccccaattgtcacagacaccagtatcagcagcagtggtggtgaaag
ctctgaagaactcagtgaaagcagctttttttaacctcatcaccccaggcgcctggaacac
tgaaaccccgcgctctagtacgcccatccccgggaccagttcaggagaatcatttgtcgg
aagcccagtttcctccgaagttgtagctgcatcgtgggaagaagccttctacacaccttt
ggcagaccagtttcgtgaactgttagttggggttgattatgtgtgggacggtgtaagggg
tttacctgtgtgttgtgtgcagcatattaacaatagtgggggaggcttgggactttgtcc
ccattgcattaatgtaggggcttggtataatggatggaaatttcgagaatttaccccaga
tttggtgcggtgtagctgccatgtgggagcttctaatcccttttctgtgctaacctgcaa
aaaatgtgcttacttgtctggattgcaaagctttgtagattatgagtaaagaaagtggca
aatggtgggaaagtgatgataaatttgctaaagctgtatatcagcaatttgtggaatttt
atgagaagcttactggaacagacttagagcttattcaaatattaaaagatcattacaata
tttctttagataatcccctagaaaacccatcttctctgtttgacttagttgctcgtatta
aaaataaccttaaaaactctccagacttatatagtcatcattttcaaagtcatggacagt
tatctgaccacccccatgccttatcatccagtaacagtcatgcagaacctagaggagaaa
atgcagtattatctagtgaagacttacacaagcctgggcaagttagcgtacaactacccg
gtactaactatgttgggcctggcaatgagctacaagctgggcccccgcaaagtgctgttg
acagtgctgcaaggattcatgactttaggtatagccaactggctaagttgggaataaatc
catatactcattggacggtagcagatgaaga
>551188
aaactgggcaataaactacacttttgatttccctggaattaatgcagatgccctccaccc
agacctccaaaccaccccaattgttacagacaccagtatcagcagcagtggtggtgaaag
ctctgaagaactcagtgaaagcagctttttttaacctcatcaccccaggcgcctggaacac
tgaaaccccgcgctctagtacgcccatccccgggaccagttcaggagaatcatttgtcgg
aagcccagtttcctccgaagttgtagctgcatcgtgggaagaagccttctacacaccttt
ggcagaccagtttcgtgaactgttagttggggttgattatgtgtgggacggtgtaagggg
tttacctgtgtgttgtgtgcagcatattaacaatagtgggggaggcttgggactttgtcc
ccattgcattaatgtaggggcttggtataatggatggaaatttcgagaatttaccccaga

tttggtgcggtgtagctgccatgtgggagcttctaatcccttttctgtgctaacctgcaa
aaaatgtgcttacttgtctggattgcaaagctttgtagattatgagtaaaaaaagtggca
aatggtgggaaagtgatgataaatttgctaaagctgtatatcagcaatttgtggaatttt
atgagaagcttactggaacagacttagagcttattcaaatattaaaagatcattacaata
tttctttagataatcccctagaaaacccatcttctctgtttgacttagttgctcgtatta
aaaataaccttaaaaactctccagacttatatagtcatcattttcaaagtcatggacagt
tatctgaccacccccatgccttatcatccagtaacagtcatgcagaacctagaggagaaa
atgcagtattatctagtgaagacttacacaagcctgggcaagttagcgtacaactacccg
gtactaactatgttgggcctggcaatgagctacaagctgggcccccgcaaagtgctgttg
acagtgctgcaaggattcatgactttaggtatagccaactggctaagttgggaataaatc
catatactcattggacggtagcagatgaaga
>712072
aaactgggcaataaactacacttttgatttccctggaattaatgcagatgccctccaccc
agacctccaaaccaccccaattgtcacagacaccagtatcagcagcagtggtggtgaaag
ctctgaagaactcagtgaaagcagctttttaacctcatcaccccaggcgcctggaacac
tgaaaccccgcgctctagtacgcccatccccgggaccagttcaggagaatcatttgtcgg
aagcccagtttcctccgaagttgtagctgcatcgtgggaagaagccttctacacaccttt
ggcagaccagtttcgtgaactgttagttggggttgattatgtgtgggacggtgtaagggg
tttacctgtgtgttgtgtgcagcatattaacaatagtgggggaggcttgggactttgtcc
ccattgcattaatgtaggggcttggtataatggatggaaatttcgagaatttaccccaga
tttggtgcggtgtagctgccatgtgggagcttctaatcccttttctgtgctaacctgcaa
aaaatgtgcttacttgtctggattgcaaagctttgtagattatgagtaaagaaagtggca
aatggtgggaaagtgatgataaatttgctaaagctgtatatcagcaatttgtggaatttt
atgagaagcttactggaacagacttagagcttattcaaatattaaaagatcattacaata
tttctttagataatcccctagaaaacccatcttctctgtttgacttagttgctcgtatta
aaaataaccttaaaaactctccagacttatatagtcatcattttcaaagtcatggacagt
tatctgaccacccccatgccttatcatccagtaacagtcatgcagaacctagaggagaaa
atgcagtattatctagtgaagacttacacaagcctgggcaagttagcgtacaactacccg
gtactaactatgttgggcctggcaatgagctacaagctgggcccccgcaaagtgctgttg
acagtgctgcaaggattcatgactttaggtatagccaactggctaagttgggaataaatc
catatactcattggacggtagcagatgaaga
>495975
aaactgggcaataaactacacttttgatttccctggaattaatgcagatgccctccaccc
agacctccaaaccaccccaattgtcacagacaccagtatcagcagcagtggtggtgaaag
ctctgaagaactcagtgaaagcagctttttaacctcatcaccccaggcgcctggaacac
tgaaaccccgcgctctagtacgcccatccccgggaccagttcaggagaatcatttgtcgg
aagcccagtttcctccgaagttgtagctgcatcgtgggaagaagccttctacacaccttt
ggcagaccagtttcgtgaactgttagttggggttgattatgtgtgggacggtgtaagggg
tttacctgtgtgttgtgtgcagcatattaacaatagtgggggaggcttgggactttgtcc
ccattgcattaatgtaggggcttggtataatggatggaaatttcgagaatttaccccaga
tttggtgcggtgtagctgccatgtgggagcttctaatcccttttctgtgctaacctgcaa
aaaatgtgcttacctgtctggattgcaaagctttgtagattatgagtaaagaaagtggca
aatggtgggaaagtgatgataaatttgctaaagctgtgtatcagcaatttgtggaatttt
atgaaaagcttactggaacagacttagagcttattcaaatattaaaagatcattacaata
tttctttagataatcccctagaaaacccatcctctctgtttgacttagttgctcgtatta
aaaataactttaaaaactctccagacttatatagtcatcattttcaaagtcatggacagt
tatctgaccacccccatgccttatcatccagtaacagtcatgcagaacctagaggagaaa

atgcagtattatctagtgaaaacttacacaagcctgggcaagttagcgtacaactacccg
gtactaactatgttgggcctggcaatgagctacaagctgggcccccgcaaagtgctgttg
acagtgctgcaaggattcatgactttaggtatagccaactggctaagttgggaataaatc
catatactcattggacggtagcagatgaaga
>728
aaactgggcaataaactacacttttgatttccctggaattaatgcagatgccctccaccc
agacctccaaaccaccccaattgtcacagacaccagtatcagcagcagtggtggtgaaag
ctctgaagaactcagtgaaagcagctttttaacctcatcaccccaggcgcctggaacac
tgaaaccccgcgctctagtacgcccatccccgggaccagttcaggagaatcatttgtcgg
aagcccagtttcctccgaagttgtagctgcatcgtgggaagaagccttctacacaccttt
ggcagaccagtttcgtgaactgttagttggggttgattatgtgtgggacggtgtaagggg
tttacctgtgtgttgtgtgcagcatattaacaatagtgggggaggcttgggactttgtcc
ccattgcattaatgtaggggcttggtataatggatggaaatttcgagaatttaccccaga
tttggtgcggtgtagctgccatgtgggagcttctaatcccttttctgtgctaacctgcaa
aaaatgtgcttacttgtctggattgcaaagctttgtagattatgagtaaagaaagtggca
aatggtgggaaagtgatgataaatttgctaaagctgtatatcagcaatttgtggaatttt
atgagaagcttactggaacagacttagagcttattcaaatattaaaagatcattacaata
tttctttagataatcccctagaaaacccatcttctctgtttgacttagttgctcgtatta
aaaataaccttaaaaactctccagacttatatagtcatcattttcaaagtcatggacagt
tatctgaccacccccatgccttatcatccagtaacagtcatgcagaacctagaggagaaa
atgcagtattatctagtgaagacttacacaagcctgggcaagttagcgtacaactacccg
gtactaactatgttgggcctggcaatgagctacaagctgggcccccgcaaagtgctgttg
acagtgctgcaaggattcatgactttaggtatagccaactggctaagttgggaataaatc
catatactcattggacggtagcagatgaaga
>389539
aaactgggcaataaactacacttttgatttccctggaattaatgcagatgccctccaccc
agacctccaaaccaccccaattgtcacagacaccagtatcagcagcagtggtggtgaaag
ctctgaagaactcagtgaaagcagctttttaacctcatcaccccaggcgcctggaacac
tgaaaccccgcgctctagtacgcccatccccgggaccagttcaggagaatcatttgtcgg
aagcccagtttcctccgaagttgtagctgcatcgtgggaagaagccttctacacaccttt
ggcagaccagtttcgtgaactgttagttggggttgattatgtgtgggacggtgtaagggg
tttacctgtgtgttgtgtgcagcatattaacaatagtgggggaggcttgggactttgtcc
ccattgcattaatgtaggggcttggtataatggatggaaatttcgagaatttaccccaga
tttggtgcggtgtagctgccatgtgggagcttctaatcccttttctgtgctaacctgcaa
aaaatgtgcttacctgtctgggttgcaaagctttgtagattatgagtaaagaaagtggca
aatggtgggaaagtgatgataaatttgctaaagctgtgtatcagcaatttgtggaatttt
atgaaaagcttactggaacagacttagagcttattcaaatattaaaagatcattacaata
tttctttagataatcccctagaaaacccatcctctctgtttgacttagttgctcgtatta
aaaataaccttaaaaactctccagacttatatagtcatcattttcaaagtcatggacagt
tatctgaccacccccatgccttatcatccagtaacagtcatgcagaacctagaggagaaa
atgcagtattatctagtgaagacttacacaagcctgggcaagttagcgtacaactacccg
gtactaactatgttgggcctggcaatgagctacaagctgggcccccgcaaagtgctgttg
acagtgctgcaaggattcatgactttaggtatagccaactggctaagttgggaataaatc
catatactcattggactgtagcagatgaaga
>411864
aaactgggcaataaactacacttttgatttccctggaattaatgcagatgccctccaccc
agacctccaaaccaccccaattgtcacagacaccagtatcagcagcagtggtggtgaaag

ctctgaagaactcagtgaaagcagctttttaacctcatcaccccaggcgcctggaacac
tgaaaccccgcgctctagtacgcccatccccgggaccagttcaggagaatcatttgtcgg
aagcccagtttcctccgaagttgtagctgcatcgtgggaagaagccttctacacaccttt
ggcagaccagtttcgtgaactgttagttggggttgattatgtgtgggacggtgtaagggg
tttacctgtgtgttgtgtgcagcatattaacaatagtgggggaggcttgggactttgtcc
ccattgcattaatgtaggggcttggtataatggatggaaatttcgagaatttaccccaga
tttggtgcggtgtagctgccatgtgggagcttctaatccctttctgtgctaacctgcaa
aaaatgtgcttacctgtctgggttgcaaagctttgtagattatgagtaaagaaagtggca
aatggtgggaaagtgatgataaatttgctaaagctgtgtatcagcaatttgtggaatttt
atgaaaagcttactggaacagacttagagcttattcaaatattaaaagatcattacaata
tttctttagataatcccctagaaaacccatcctctctgtttgacttagttgctcgtatta
aaaataaccttaaaaactctccagacttatatagtcatcattttcaaagtcatggacagt
tatctgaccacccccatgccttatcatccagtaacagtcatgcagaacctagaggagaaa
atgcagtattatctagtgaagacttacacaagcctgggcaagttagcgtacaactacccg
gtactaactatgttgggcctggcaatgagctacaagctgggcccccgcaaagtgctgttg
acagtgctgcaaggattcatgactttaggtatagccaactggctaagttgggaataaatc
catatactcattggactgtagcagatgaaga
>314918
aaactgggcaataaactacacttttgatttccctggaattaatacagatgccctccaccc
agacctccaaaccaccccaattgtcacagacaccagtatcagcagcagtggtggtgaaag
ctctgaagaactcagtgaaagcagctttttaacctcatcaccccaggcgcctggaacac
tgaaaccccgcgctctagtacgcccatccccgggaccagttcaggagaatcatttgtcgg
aagcccagtttcctccgaagttgtagctgcatcgtgggaagaagccttctacacaccttt
ggcagaccagtttcgtgaactgttagttggggttgattatgtgtgggacggtgtaagggg
tttacctgtgtgttgtgtgcagcatattaacaatagtgggggaggcttgggactttgtcc
ccattgcattaatgtaggggcttggtataatggatggaaatttcgagaatttaccccaga
tttggtgcggtgtagctgccatgtgggagcttctaatccctttctgtgctaacctgcaa
aaaatgtgcttacctgtctggattgcaaagctttgtagattatgagtaaagaaagtggca
aatggtgggaaagtgatgataaatttgctaaagctgtgtatcagcaatttgtggaatttt
atgaaaagcttactggaacagacttagagcttattcaaatattaaaagatcattacaata
tttctttagataatcccctagaaaacccatcctctctgtttgacttagttgctcgtatta
aaaataaccttaaaaactctccagacttatatagtcatcattttcaaagtcatggacagt
tatctgaccacccccatgccttatcatccagtaacagtcatgcagaacctagaggagaaa
atgcagtattatctagtgaagacttacacaagcctgggcaagttagcgtacaactacccg
gtactaactatgttgggcctggcaatgagctacaagctgggcccccgcaaagtgctgttg
acagtgctgcaaggattcatgactttaggtatagccaactggctaagttgggaataaatc
catatactcattggactgtagcagatgaaga
>745152
aaactgggcaataaactacacttttgatttccctggaattaatgcagatgccctccaccc
agacctccaaaccaccccaattgtcacagacaccagtatcagcagcagtggtggtgaaag
ctctgaagaactcagtgaaagcagctttttaacctcatcaccccaggcgcctggaacac
tgaaaccccgcgctctagtacgcccatccccgggaccagttcaggagaatcatttgtcgg
aagcccagtttcctccgaagttgtagctgcatcgtgggaagaagccttctacacaccttt
ggcagaccagtttcgtgaactgttagttggggttgattatgtgtgggacggtgtaagggg
tttacctgtgtgttgtgtgcagcatattaacaatagtgggggaggcttgggactttgtcc
ccattgcattaatgtaggggcttggtataatggatggaaatttcgagaatttaccccaga
tttggtgcggtgtagctgccatgtgggagcttctaatccctttctgtgctaacctgcaa

aaaatgtgcttacttgtctggattgcaaagctttgtagattatgagtaaagaaagtggca
aatggtgggaaagtgatgataaatttgctaaagctgtatatcagcaatttgtggaatttt
atgagaagcttactggaacagacttagagcttattcaaatattaaaagatcattacaata
tttctttagataatcccctagaaaacccatcttctctgtttgacttagttgctcgtatta
aaaataaccttaaaaactctccagacttatatagtcatcattttcaaagtcatggacagt
tatctgaccacccccatgccttatcatccagtaacagtcatgcagaacctagaggagaaa
atgcagtattatctagtgaagacttacacaagcctgggcaagttagcgtacaactacccg
gtactaactatgttgggcctggcaatgagctacaagctgggcccccgcaaagtgctgttg
acagtgctgcaaggattcatgactttaggtatagccaactggctaagttgggaataaatc
catatactcattggactgtagcagatgaaga
>634558
aaactgggcaataaactacacttttgatttccctggaattaatgcagatgccctccaccc
agacctccaaaccactccaattgtcacagacaccagtatcagcagcagtggtggtgaaag
ctctgaagaactcagtgaaagcagctttttaacctcatcaccccaggcgcctggaacac
tgaaaccccgcgctctagtacgcccatccccgggaccagttcaggagaatcatttgtcgg
aagcccagtttcctccgaagttgtagctgcatcgtgggaagaagccttctacacaccttt
ggcagaccagtttcgtgaactgttagttggggttgattatgtgtgggacggtgtaagggg
tttacctgtgtgttgtgtgcagcatattaacaatagtgggggaggcttgggactttgtcc
ccattgcattaatgtaggggcttggtataatggatggaaatttcgagaatttaccccaga
tttggtgcggtgtagctgccatgtgggagcttctaatccctttctgtgctaacctgcaa
aaaatgtgcttacctgtctgggttgcaaagctttgtagattatgagtaaagaaagtggca
aatggtgggaaagtgatgataaatttgctaaagctgtgtatcagcaatttgtggaatttt
atgaaaagcttactggaacagacttagagcttattcaaatattaaaagatcattacaata
tttctttagataatcccctagaaaacccatcctctctgtttgacttagttgctcgtatta
aaaataaccttaaaaactctccagacttatatagtcatcattttcaaagtcatggacagt
tatctgaccacccccatgccttatcatccagtaacagtcatgcagaacctagaggagaaa
atgcagtattatctagtgaagacttacacaagcctgggcaagttagcgtacaactacccg
gtactaactatgttgggcctggcaatgagctacaagctgggcccccgcaaagtgctgttg
acagtgctgcaaggattcatgactttaggtatagccaactggctaagttgggaataaatc
catatactcattggacggtagcagatgaaga
>642423
aaactgggcaataaactacacttttgatttccctggaattaatgcagatgccctccaccc
agacctccaaaccaccccaattgtcacagacaccagtatcagcagcagtggtggtgaaag
ctctgaagaactcagtgaaagcagctttttaacctcatcaccccaggcgcctggaacac
tgaaaccccgcgctctagtacgcccatccccgggaccagttcaggagaatcatttgtcgg
aagcccagtttcctccgaagttgtagctgcatcgtgggaagaagccttctacacaccttt
ggcagaccagtttcgtgaactgttagttggggttgattatgtgtgggacggtgtaagggg
tttacctgtgtgttgtgtgcagcatattaacaatagtgggggaggcttgggactttgtcc
ccattgcattaatgtaggggcttggtataatggatggaaatttcgagaatttaccccaga
tttggtgcggtgtagctgccatgtgggagcttctaatccctttctgtgctaacctgcaa
aaaatgtgcttacctgtctgggttgcaaagctttgtagattatgagtaaagaaagtggca
aatggtgggaaagtgatgataaatttgctaaagctgtgtatcagcaatttgtggaatttt
atgaaaagcttactggaacagacttagagcttattcaaatattaaaagatcattacaata
tttctttagataatcccctagaaaacccatcctctctgtttgacttagttgctcgtatta
aaaataaccttaaaaactctccagacttatatagtcatcattttcaaagtcatggacagt
tatctgaccacccccatgccttatcatccagtaacagtcatgcagaacctagaggagaaa
atgcagtattatctagtgaagacttacacaagcctgggcaagttagcgtacaactacccg

gtactaactatgttgggcctggcaatgagctacaagctgggcccccgcaaagtgctgttg
acagtgctgcaaggattcatgactttaggtatagccaactggctaagttgggaataaatc
catatactcattggacggtagcagatgaaga
>339097
aaactgggcaataaactacacttttgatttccctggaattaatgcagatgcccttcaccc
agacctccaaaccaccccaattgtcacagacaccagtatcagcagcagtggtggtgaaag
ctctgaagaactcagtgaaagcagctttttaacctcatcaccccaggcgcctggaacac
tgaaaccccgcgctctagtacgcccatccccgggaccagttcaggagaatcatttgtcgg
aagcccagtttcctccgaagttgtagctgcatcgtgggaagaagccttctacacaccttt
ggaagaccagtttcgtgaactgttagttggggttgattatgtgtgggacggtgtaagggg
tttacctgtgtgttgtgtgcagcatattaacaatagtgggggaggcttgggactttgtcc
ccattgcattaatgtaggggcttggtataatggatggaaatttcgagaatttaccccaga
tttggtgcggtgtagctgccatgtgggagcttctaatcccttttctgtgctaacctgcaa
aaaatgtgcttacctgtctggattgcaaagctttgtagattatgagtaaagaaagtggca
aatggtgggaaagtgatgataaatttgctaaagctgtgtatcagcaatttgtggaatttt
atgaaaagcttactggaacagacttagagcttattcaaatattaaaagatcattacaata
tttctttagataatcccctagaaaacccatcctctctgtttgacttagttgctcgtatta
aaaataaccttaaaaactctccagacttatatagtcatcattttcaaagtcatggacagt
tatctgaccaccccatgccttatcatccagtaacagtcatgcagaacctagaggagaaa
atgcagtattatctagtgaagacttacacaagcctgggcaagttagcgtacaactacccg
gtactaactatgttgggcctggcaatgagctacaagctgggcccccgcaaagtgctgttg
acagtgctgcaaggattcatgactttaggtatagccaactggctaagttgggaataaatc
catatactcattggacggtagcagatgaaga
>284066
aaactgggcaataaactacacttttgatttccctggaattaatgcagatgccctccaccc
agacctccaaaccaccccaattgtcacagacaccagtatcagcagcagtggtggtgaaag
ctctgaagaactcagtgaaagcagctttttaacctcatcaccccaggcgcctggaacac
tgaaaccccgcgctctagtacgcccatccccgggaccagttcaggagaatcacttgtcgg
aagcccagtttcctccgaagttgtagctgcatcgtgggaagaagccttctacacaccttt
ggcagaccagtttcgtgaactgttagttggggttgattatgtgtgggacggtgtaagggg
tttacctgtgtgctgtgtgcaacatattaacaatagtgggggaggcttgggactttgtcc
ccattgcattaatgtaggggcttggtataatggatggaaatttcgagaatttaccccaga
tttggtgcgatgtagctgccatgtgggagcttctaatcccttttctgtgctaacctgcaa
aaaatgtgcttacctgtctggattgcaaagctttgtagattatgagtaaagaaagtggca
aatggtgggaaagtgatgataaatttgctaaagctgtgtatcagcaatttgtggaatttt
atgaaaaggttactggaacagacttagagcttattcaaatattaaaagatcattataata
tttctttagataatcccctagaaaacccatcctctctgtttgacttagttgctcgtatta
aaaataaccttaaaaactctccagacttatatagtcatcattttcaaagtcatggacagt
tatctgaccacccccatgccttatcatccagtagcagtcatgcagaacctagaggagaaa
atgcagtattatctagtgaagacttacacaagcctgggcaagttagcgtacaactacccg
gtactaactatgttgggcctggcaatgagctacaagctgggcccccgcaaagtgctgttg
acagtgctgcaaggattcatgactttaggtatagccaactggctaagttgggaataaatc
catatactcattggacggtagcagatgaaga
>318400
aaactgggcaataaactacacttttgatttccctggaattaatgcagatgccctccaccc
agacctccaaaccaccccaattgtcacagacaccagtatcagcagcagtggtggtgaaag
ctctgaagaactcagtgaaagcagctttcttaacctcatcaccccaggcgcctggaacac

tgaaaccccgcgctctagtacgcccatccccgggaccagttcaggagaatcatctgtcgg
aagcccagtttcctccgaagttgtagctgcatcgtgggaagaagccttctacacaccttt
ggcagaccagtttcgtgaactgttagttggggttgattatgtgtgggacggtgtaagggg
tttacctgtgtgttgtgtgcaacatattaacaatagtgggggaggcttgggactttgtcc
ccattgcattaatgtaggggcttggtataatggatggaaatttcgagaatttaccccaga
tttggtgcggtgtagctgccatgtgggagcttctaatcccttttctgtgctaacctgcaa
aaaatgtgcttacttgtctggattgcaaagctttgtagattatgagtaaagaaagtggca
aatggtgggaaagtgatgataaatttgctaaagctgtgtatcagcaatttgtggaatttt
atgaaaaggttactggtacagacttagagcttattcaaatattaaaagatcattataata
tttctttagataatcccctagaaaacccatcctctctgtttgacttagttgctcgtatta
aaaataaccttaaaaactctccagacttatatagtcatcattttcaaagtcatggacact
tatctgaccacccccatgccttatcatccagtagcagtcatgcagaacctagaggagaaa
atgcagtattatctagtgaagacttacacaagcctgggcaagttagcgtacaactacccg
gtactaactatgttgggcctggcaatgagctacaagctgggcccccgcaaagtgctgttg
acagtgctgcaaggattcatgactttaggtatagccaactggctaagttgggaataaatc
catatactcattggacggtagcagatgaaga
>390408
aaactgggcaataaactacacttttgatttccctggaattaatgcagatgccctccaccc
agacctccaaaccaccccaattgtcacagacaccagtatcagcagcagtggtggtgaaag
ctctgaagaactcagtgaaagcagctttttaacctcatcaccccaggcgcctggaacac
tgaaaccccgcgctctagtacgcccatccccgggaccagttcaggagaatcacttgtcgg
aagcccagtttcctccgaagttgtagctgcatcgtgggaagaagccttctacacaccttt
ggcagaccagtttcgtgaactgttagttggggttgattatgtgtgggacggtgtaagggg
tttacctgtgtgttgtgtgcaacatattaacaatagtgggggaggcttgggactttgtcc
ccattgcattaatgtaggggcttggtataatggatggaaatttcgagaattcaccccaga
tttggtgcggtgtagctgccatgtgggagcttctaatcccttttctgtgctaacctgcaa
aaaatgtgcttacctgtctggattgcaaagctttgtagattatgagtaaagaaagtggca
aatggtgggaaagtgatgataaatttgctaaagctgtgtatcagcaatttgtggaatttt
atgaaaaggttactggaacagacttagagcttattcaaatattaaaagatcattacaata
tttctttagataacccccctagaaaatccatcctctctgtttgacttagttgctcgtatta
aaaataaccttaaaaactctccagacttatatagtcatcattttcaaagtcatggacagt
tatctgaccacccccatgccttatcatccagtgacagtcatgcagaacctagaggagaag
atgcagtattatctagtgaagacttacacaagcctgggcaagttagcgtacaactacccg
gtactaactatgttgggcctggcaatgagctacaagctgggcccccgcaaagtgctgttg
acagtgctgcaaggattcatgactttaggtatagccaactggctaagttgggaataaatc
catatactcattggacggtagcagatgaaga
>454053
aaactgggcaataaactacacttttgatttccctggaattaatgcagatgccctccaccc
agacctccaaaccactccaattgtcacagacaccagtatcagcagcagtggtggtgaaag
ctctgaagaactcagtgaaagcagctttttaacctcatcaccccaggcgcctggaacac
tgaaaccccgcgctctagtacgcccatccccgggaccagttcaggagaatcatttgtcgg
aagcccagtttcctccgaagttgtagctgcatcgtgggaagaagccttctacacaccttt
ggcagaccagtttcgtgaactgttagttggggttgattatgtgtgggacggtgtaagggg
tttacctgtgtgttgtgtgcagcatattaacaatagtgggggaggcttgggactttgtcc
ccattgcattaatgtaggggcttggtataatggatggaaatttcgagaatttaccccaga
tttggtgcggtgtagctgccatgtgggagcttctaatcccttttctgtgctaacctgcaa
aaaatgtgcttacctgtctgggttgcaaagctttgtagattatgagtaaagaaagtggca

aatggtgggaaagtgatgataaatttgctaaagctgtgtatcagcaatttgtggaatttt
atgaaaagcttactggaacagacttagagcttattcaaatattaaaagatcattacaata
tttctttagataatcccctagaaaacccatcctctctgtttgacttagttgctcgtatta
aaaataaccttaaaaactctccagacttatatagtcatcattttcaaagtcatggacagt
tatctgaccacccccatgccttatcatccagtaacagtcatgcagaacctagaggagaaa
atgcagtattatctagtgaagacttacacaagcctgggcaagttagcgtacaactacccg
gtactaactatgttgggcctggcaatgagctacaagctgggcccccgcaaagtgctgttg
acagtgctgcaaggattcatgactttaggtatagccaactggctaagttgggaataaatc
catatactcattggacggtagcagatgaaga
>496304
aaactgggcaataaactacacttttgatttccctggaattaatgcagatgccctccaccc
agacctccaaaccactccaattgtcacagacaccagtatcagcagcagtggtggtgaaag
ctctgaagaactcagtgaaagcagctttttaacctcatcaccccaggcgcctggaacac
tgaaaccccgcgctctagtacgcccatccccgggaccagttcaggagaatcatttgtcgg
aagcccagtttcctccgaagttgtagctgcatcgtgggaagaagccttctacacaccttt
ggcagaccagtttcgtgaactgttagttggggttgattatgtgtgggacggtgtaagggg
tttacctgtgtgttgtgtgcagcatattaacaatagtgggggaggcttgggactttgtcc
ccattgcattaatgtaggggcttggtataatggatggaaatttcgagaatttaccccaga
tttggtgcggtgtagctgccatgtgggagcttctaatcccttttctgtgctaacctgcaa
aaaatgtgcttacctgtctgggttgcaaagctttgtagattatgagtaaagaaagtggca
aatggtgggaaagtgatgataaatttgctaaagctgtgtatcagcaatttgtggaatttt
atgaaaagcttactggaacagacttagagcttattcacatattaaaacatcattacaata
tttctttagataatcccctagaaaacccatcctctctgtttgacttagttgctcgtatta
aaaataaccttaaaaactctccagacttatatagtcatcattttcaaagtcatggacagt
tatctgaccacccccatgccttatcatccagtaacagtcatgcagaacctagaggagaaa
atgcagtattatctagtgaagacttacacaagcctgggcaagttagcgtacaactacccg
gtactaactatgttgggcctggcaatgagctacaagctgggcccccgcaaagtgctgttg
acagtgctgcaaggattcatgactttaggtatagccaactggctaagttgggaataaatc
catatactcattggactgtagcagatgaaga
>731921
aaactgggcaataaactacacttttgatttccctggaattaatgcagatgccctccaccc
agacctccaaaccaccccaattgtcacagacaccagtatcagcagcagtggtggtgaaag
ctctgaagaactcagtgaaagcagctttttaacctcatcaccccaggcgcctggaacac
tgaaaccccgcgctctagtacgcccatccccgggaccagttcaggagaatcatttgtcgg
aagcccagtttcctccgaagttgtagctgcatcgtgggaagaagccttctacacaccttt
ggcagaccagtttcgtgaactgttagttggggttgattatgtgtgggacggtgtaagggg
tttacctgtgtgttgtgtgcagcatattaacaatagtgggggaggcttgggactttgtcc
ccattgcattaatgtaggggcttggtataatggatggaaatttcgagaatttaccccaga
tttggtgcggtgtagctgccatgtgggagcttctaatcccttttctgtgctaacctgcaa
aaaatgtgcttacttgtctggattgcaaagctttgtagattatgagtaaagaaagtggca
aatggtgggaaagtgatgataaatttgctaaagctgtatatcagcaatttgtggaatttt
atgagaagcttactggaacagacttagagcttattcaaatattaaaagatcattacaata
tttctttagataatcccctagaaaacccatcttctctgtttgacttagttgctcgtatta
aaaataaccttaaaaactctccagacttatatagtcatcattttcaaagtcatggacagt
tatctgaccacccccatgccttatcatccagtaacagtcatgcagaacctagaggagaaa
atgcagtattatctagtgaagacttacacaagcctgggcaagttagcgtacaactacccg
gtactaactatgttgggcctggcaatgagctacaagctgggcccccgcaaagtgctgttg

acagtgctgcaaggattcatgactttaggtatagccaactggctaagttgggaataaatc
catatactcattggactgtagcagatgaaga
>551061
aaactgggcaataaactacacttttgatttccctggaattaatgcagatgccctccaccc
agacctccaaaccaccccaattgtcacagacaccagtatcagcagcagtggtggtgaaag
ctctgaagaactcagtgaaagcagcttttttaacctcatcaccccaggcgcctggaacac
tgaaaccccgcgctctagtacgcccatccccgggaccagttcaggagaatcatttgtcgg
aagcccagtttcctccgaagttgtagctgcatcgtgggaagaagccttctacacaccttt
ggcagaccagtttcgtgaactgttagttggggttgattatgtgtgggacggtgtaagggg
tttacctgtgtgttgtgtgcagcatattaacaatagtgggggaggcttgggactttgtcc
ccattgcattaatgtaggggcttggtataatggatggaaatttcgagaatttaccccaga
tttggtgcggtgtagctgccatgtgggagcttctaatcccttttctgtgctaacctgcaa
aaaatgtgcttacttgtctggattgcaaagctttgtagattatgagtaaagaaagtggca
aatggtgggaaagtgatgataaatttgctaaagctgtatatcagcaatttgtggaatttt
atgagaagcttactggaacagacttagagcttattcaaatattaaaagatcattacaata
tttctttagataatcccctagaaaacccatcttctctgtttgacttagttgctcgtatta
aaaataaccttaaaaactctccagacttatatagtcatcattttcaaagtcatggacagt
tatctgaccacccccatgccttatcatccagtaacagtcatgcagaacctagaggagaaa
atgcagtattatctagtgaagacttacacaagcctgggcaagttagcgtacaactacccg
gtactaactatgttgggcctggcaatgagctacaagctgggcccccgcaaagtgctgttg
acagtgctgcaaggattcatgactttaggtatagccaactggctaagttgggaataaatc
catatactcattggactgtagcagatgaaga
>745241
aaactgggcaataaactacacttttgatttccctggaattaatgcagatgccctccaccc
agacctccaaaccaccccaattgtcacagacaccagtatcagcagcagtggtggtgaaag
ctctgaagaactcagtgaaagcagcttttttaacctcatcaccccaggcgcctggaacac
tgaaaccccgcgctctagtacgcccatccccgggaccagttcaggagaatcatttgtcgg
aagcccagtttcctccgaagttgtagctgcatcgtgggaagaagccttctacacaccttt
ggcagaccagtttcgtgaactgttagttggggttgattatgtgtgggacggtgtaagggg
tttacctgtgtgttgtgtgcagcatattaacaatagtgggggaggcttgggactttgtcc
ccattgcattaatgtaggggcttggtataatggatggaaatttcgagaatttaccccaga
tttggtgcggtgtagctgccatgtgggagcttctaatcccttttctgtgctaacctgcaa
aaaatgtgcttacttgtctggattgcaaagctttgtagattatgagtaaagaaagtggca
aatggtgggaaagtgatgataaatttgctaaagctgtatatcagcaatttgtggaatttt
atgagaagcttactggaacagacttagagcttattcaaatattaaaagatcattacaata
tttctttagataatcccctagaaaacccatcttctctgtttgacttagttgctcgtatta
aaaataaccttaaaaactctccagacttatatagtcatcattttcaaagtcatggacagt
tatctgaccacccccatgccttatcatccagtaacagtcatgcagaacctagaggagaaa
atgcagtattatctagtgaagacttacacaagcctgggcaagttagcgtacaactacccg
gtactaactatgttgggcctggcaatgagctacaagctgggcccccgcaaagtgctgttg
acagtgctgcaaggattcatgactttaggtatagccaactggctaagttgggaataaatc
catatactcattggactgtagcagatgaaga
>739565
aaactgggcaataaactacacttttgatttccctggaattaatgcagatgccctccaccc
agacctccaaaccaccccaattgtcacagacaccagtatcagcagcagtggtggtgaaag
ctctgaagaactcagtgaaagcagcttttttaacctcatcaccccaggcgcctggaacac
tgaaaccccgcgctctagtacgcccatccccgggaccagttcaggagaatcatttgtcgg

aagcccagtttcctccgaagttgtagctgcatcgtgggaagaagccttctacacaccttt
ggcagaccagtttcgtgaactgttagttggggttgattatgtgtgggacggtgtaagggg
tttacctgtgtgttgtgtgcagcatattaacaatagtgggggaggcttgggactttgtcc
ccattgcattaatgtaggggcttggtataatggatggaaatttcgagaatttaccccaga
tttggtgcggtgtagctgccatgtgggagcttctaatcccttttctgtgttaacctgcaa
aaaatgtgcttacttgtctggattgcaaagctttgtagattatgagtaaagaaagtggca
aatggtgggaaagtgatgataaatttgctaaagctgtatatcagcaatttgtggaatttt
atgagaagcttactggaacagacttagagcttattcaaatattaaaagatcattacaata
tttctttagataatcccctagaaaacccatcttctctgtttgacttagttgctcgtatta
aaaataaccttaaaaactctccagacttatatagtcatcattttcaaagtcatggacagt
tatctgaccacccccatgccttatcatccagtaacagtcatgcagaacctagaggagaaa
atgcagtattatctagtgaagacttacacaagcctgggcaagttagcgtacaactacccg
gtactaactatgttgggcctggcaatgagctacaagctgggcccccgcaaagtgctgttg
acagtgctgcaaggattcatgactttaggtatagccaactggctaagttgggaataaatc
catatactcattggactgtagcagatgaaga
>366548
aaactgggcaataaactacacttttgatttccctggaattaatgcagatgccctccaccc
agacctccaaaccaccccaattgtcacagacaccagtatcagcagcagtggtggtgaaag
ctctgaagaactcagtgaaagcagctttttaacctcatcaccccaggcgcctggaacac
tgaaaccccgcgctctagtacgcccatccccgggaccagttcaggagaatcatttgtcgg
aagcccagtttcctccgaagttgtagctgcatcgtgggaagaagccttctacacaccttt
ggcagaccagtttcgtgaactgttagttggggttgattatgtgtgggacggtgtaagggg
tttacctgtgtgttgtgtgcagcatattaacaatagtgggggaggcttgggactttgtcc
ccattgcattaatgtaggggcttggtataatggatggaaatttcgagaatttaccccaga
tttggtgcggtgtagctgccatgtgggagcttctaatcccttttctgtgctaacctgcaa
aaaatgtgcttacctgtctgggttgcaaagctttgtagattatgagtaaagaaagtggca
aatggtgggaaagtgatgataaatttgctaaagctgtgtatcagcaatttgtggaatttt
atgaaaagcttactggaacagacttagagcttattcaaatattaaaagatcattacaata
tttctttagataatcccctagaaaacccatcctctctgtttgacttagttgctcgtatta
aaaataaccttaaaaactctccagacttatatagtcatcattttcaaagtcatggacagt
tatctgaccacccccatgccttatcatccagtaacagtcatgcagaacctagaggagaaa
atgcagtattatctagtgaagacttacacaagcctgggcaagttagcgtacaactacccg
gtactaactatgttgggcctggcaatgagctacaagctgggcccccgcaaagtgctgttg
acagtgctgcaaggattcatgactttaggtatagccaactggctaagttgggaataaatc
catatactcattggactgtagcagatgaaga
>391900
aaactgggcaataaactacacttttgatttccctggaattaatgcagatgccctccaccc
agacctccaaaccaccccaattgtcacagacaccagtatcagcagcagtggtggtgaaag
ctctgaagaactcagtgaaagcagctttttaacctcatcaccccaggcgcctggaacac
tgaaaccccgcgctctagtacgcccatccccgggaccagttcaggagaatcatttgtcgg
aagcccagtttcctccgaagttgtagctgcatcgtgggaagaagccttctacacaccttt
ggcagaccagtttcgtgaactgttagttggggttgattatgtgtgggacggtgtaagggg
tttacctgtgtgttgtgtgcagcatattaacaatagtgggggaggcttgggactttgtcc
ccattgcattaatgtaggggcttggtataatggatggaaatttcgagaatttaccccaga
tttggtgcggtgtagctgccatgtgggagcttctaatcccttttctgtgctaacctgcaa
aaaatgtgcttacctgtctggattgcaaagctttgtagattatgagtaaaaaaagtggca
aatggtgggaaagtgatgataaatttgctaaagctgtgtatcagcaatttgtggaatttt

atgaaaagcttactggaacagacttagagcttattcaaatattaaaagatcattacaata
tttctttagataatcccctagaaaacccatcctctctgtttgacttagttgctcgtatta
aaaataaccttaaaaactctccagacttatatagtcatcattttcaaagtcatggacagt
tatctgaccacccccatgccttatcatccagtaacagtcatgcagaacctagaggagaaa
atgcagtattatctagtgaagacttacacaagcctgggcaagttagcgtacaactacccg
gtactaactatgttgggcctggcaatgagctacaagctgggcccccgcaaagtgctgttg
acagtgctgcaaggattcatgactttaggtatagccaactggctaagttgggaataaatc
catatactcattggactgtagcagatgaaga
>449145
aaactgggcaataaactacacttttgatttccctggaattaatgcagatgccctccaccc
agacctccaaaccactccaattgtcacagacaccagtatcagcagcagtggtggtgaaag
ctctgaagaactcagtgaaagcagctttttaacctcatcaccccaggcgcctggaacac
tgaaaccccgcgctctagtacgcccatccccgggaccagttcaggagaatcatttgtcgg
aagcccagtttcctccgaagttgtagctgcatcgtgggaagaagccttctacacaccttt
ggcagaccagtttcgtgaactgttagttggggttgattatgtgtgggacggtgtaagggg
tttacctgtgtgttgtgtgcagcatattaacaatagtgggggaggcttgggactttgtcc
ccattgcattaatgtaggggcttggtataatggatggaaatttcgagaatttaccccaga
tttggtgcggtgtagctgccatgtgggagcttctaatcccttttctgtgctaacctgcaa
aaaatgtgcttacctgtctgggttgcaaagctttgtagattatgagtaaagaaagtggca
aatggtgggaaagtgatgataaatttgctaaagctgtgtatcagcaatttgtggaatttt
atgaaaagcttactggaacagacttagagcttattcaaatattaaaagatcattacaata
tttctttagataatcccctagaaaacccatcctctctgtttgacttagttgctcgtatta
aaaataaccttaaaaactctccagacttatatagtcatcattttcaaagtcatggacagt
tatctgaccacccccatgccttatcatccagtaacagtcatgcagaacctagaggagaaa
atgcagtattatctagtgaaaacttacacaagcctgggcaagttagcgtacaactacccg
gtactaactatgttgggcctggcaatgagctacaagctgggcccccgcaaagtgctgttg
acagtgctgcaaggattcatgactttaggtatagccaactggctaagttgggaataaatc
catatactcattggactgtagcagatgaaga
>517902
aaactgggcaataaactacacttttgatttccctggaattaatgcagatgccctccaccc
agacctccaaaccactccaattgtcacagacaccagtatcagcagcagtggtggtgaaag
ctctgaagaactcagtgaaagcagctttttaacctcatcaccccaggcgcctggaacac
tgaaaccccgcgctctagtacgcccatccccgggaccagttcaggagaatcatttgtcgg
aagcccagtttcctccgaagttgtagctgcatcgtgggaagaagccttctacacaccttt
ggcagaccagtttcgtgaactgttagttggggttgattatgtgtgggacggtgtaagggg
tttacctgtgtgttgtgtgcagcatattaacaatagtgggggaggcttgggactttgtcc
ccattgcattaatgtaggggcttggtataatggatggaaatttcgagaatttaccccaga
tttggtgcggtgtagctgccatgtgggagcttctaatcccttttctgtgctaacctgcaa
aaaatgtgcttacctgtctgggttgcaaagctttgtagattatgagtaaagaaagtggca
aatggtgggaaagtgatgataaatttgctaaagctgtgtatcagcaatttgtggaatttt
atgaaaagcttactggaacagacttagagcttattcaaatattaaaagatcattacaata
tttctttagataatcccctagaaaacccatcctctctgtttgacttagttgctcgtatta
aaaataaccttaaaaactctccagacttatatagtcatcattttcaaagtcatggacagt
tatctgaccacccccatgccttatcatccagtaacagtcatgcagaacctagaggagaaa
atgcagtattatctagtgaagacttacacaagcctgggcaagttagcgtacaactacccg
gtactaactatgttgggcctggcaatgagctacaagctgggcccccgcaaagtgctgttg
acagtgctgcaaggattcatgactttaggtatagccaactggctaagttgggaataaatc

catatactcattggactgtagcagatgaaga
>352434
aaactgggcaataaactacacttttgatttccctggaattaatgcagatgccctccaccc
agacctccaaaccaccccaattgtcacagacaccagtatcagcagcagtggtggtgaaag
ctctgaagaactcagtgaaagcagctttttttaacctcatcaccccaggcgcctggaacac
tgaaaccccgcgctctagtacgcccatccccgggaccagttcaggagaatcatttgtcgg
aagcccagtttcctccgaagttgtagctgcatcgtgggaagaagccttctacacaccttt
ggcagaccagtttcgtgaactgttagttggggttgattatgtgtgggacggtgtaagggg
tttacctgtgtgttgtgtgcagcatattaacaatagtgggggaggcttgggactctgtcc
ccattgtattaatgtaggggcttggtataatggatggaaatttcgagaatttaccccaga
tttggtgcggtgcagctgccatgtgggagcttctaatcccttttctgtgctaacctgcaa
aaaatgtgcttacctgtctggattgcaaagctttgtagattatgagtaaacaaagtggca
aatggtgggaaagtgatgataaatttgctaaagctgtgtatcagcaatttgtggaatttt
atgaaaagcttactggaacagatttagagcttattcaaatattaaaagatcattacaata
tttctttagataatcccctagaaaacccatcttctctgtttgacttagttgctcgtatta
aaaataaccttaaaaactctccagacttatatagtcatcattttcaaagtcatggacagt
tatctgaccacccccatgccttatcatccagtaacagtcatgcagaacctagaggagaaa
atgcagtattatctagtgaagacttacacaagcctgggcaagttagcgtacaactacccg
gtactaactatgttgggcctggcaatgagctacaagctgggcccccgcaaagtgctgttg
acagtgctgcaaggattcatgactttaggtatagccaactggctaagttgggaataaatc
catatactcattggacggtagcagatgaaga
>289912
aaactgggcaataaactacacttttgatttccctggaattaatgcagatgccctccaccc
agacctccaaaccaccccaattgtcacagacaccagtatcagcagcagtggtggtgaaag
ctctgaagaactcagtgaaagcagctttttttaacctcatcaccccaggcgcctggaacac
tgaaaccccgcgctctagtacgcccatccccgggaccagttcaggagaatcacttgtcgg
aagcccagtttcctccgaagttgtagctgcatcgtgggaagaagccttctacacaccttt
ggcagaccagtttcgtgaactgttagttggggttgattatgtgtgggacggtgtaagggg
tttacctgtgtgttgtgttcagcatattaacaatagtgggggaggcttgggactttgtcc
ccattgcattaatgtaggggcttggtataatggatggaaatttcgagaatttaccccaga
tttggtgcggtgtagctgccatgtgggagcttctaatcccttttctgtgctaacctgcaa
aaaatgtgcttacctatctggattgcaaagctttgtagattatgagtaaagaaagtggca
aatggtgggaaagtgatgataaatttgctaaagctgtgtatcagcaatttgtggaatttt
atgaaaagcttactggaacagacttagagcttattcaaatattaaaagatcattacaata
tttctttagataatcccctagaaaacccatcctctctgtttgacttagttgctcgtatta
aaaataaccttaaaaactctccagacttatatagtcatcattttcaaagtcatggacagt
tatctgaccacccccatgccttatcatccagtaacagtcatgcagaacctagaggagaaa
atgcagtattatctagtgaagacttacacaagcctgggcaagttagcgtacaactacccg
gtactaactatgttgggcctggcaatgagctacaagctgggcccccgcaaagtgctgttg
acagtgctgcaaggattcatgactttaggtatagccaactggctaagttgggaataaatc
catatactcattggacggtagcagatgaaga
>452009
aaactgggcaataaactacacttttgattttcctggaattaatgcagatgccctccaccc
agacctccaaaccaccccaattgtcacagacaccagtatcagcagcagtggtggtgaaag
ctctgaagaactcagtgaaagcagctttttttaacctcatcaccccaggcgcctggaacac
tgaaaccccgcgctctagtacgcccatccccgggaccagttcaggagaatcacttgtcgg
aagcccagtttcctccgaagttgtagctgcatcgtgggaagaagccttctacacaccttt

ggcagaccagtttcgtgaactgttagttggggttgattatgtgtgggacggtgtaagggg
tttacctgtgtgttgtgttcagcatattaacaatagtgggggaggcttgggactttgtcc
ccattgcattaatgtaggggcttggtataatggatggaaatttcgagaatttaccccaga
tttggtgcggtgtagctgccatgtgggagcttctaatcccttttctgtgctaacctgcaa
aaaatgtgcttacctatctggattgcaaagctttgtagattatgagtaaagaaagtggca
aatggtgggaaagtgatgataaatttgctaaagctgtgtatcagcaatttgtagaatttt
atgaaaagcttactggaacagacttagagcttattcaaatattaaaaaatcattacaata
tttctttagataatcccctagaaaacccatcctctctgtttgacttagttgctcgtatta
aaaataaccttaaaaactctccagacttatatagtcatcattttcaaagtcatggacagt
tatctgaccacccccatgccttatcatccagtaacagtcatgcagaacctagaggagaaa
atgcagtattatctagtgaagacttacacaagcctgggcaagttagcgtacaactacccg
gtactaactatgttgggcctggcaatgagctacaagctgggcccccgcaaagtgctgttg
acagtgctgcaaggattcatgactttaggtatagccaactggctaagttgggaataaatc
catatactcattggactgtagcagatgaaga
>509602
aaactgggcaataaactacacttttgatttccctggaattaatgcagatgccctccaccc
agacctccaaaccaccccaattgtcacagacaccagtatcagcagcagtggtggtgaaag
ctctgaagaactcagtgaaagcagctttttttaacctcatcaccccaggcgcctggaacac
tgaaaccccgcgctctagtacgcccatccccgggaccagttcaggagaatcacttgtcgg
aagcccagtttcctccgaagttgtagctgcatcgtgggaagaagccttctacacaccttt
ggcagaccagtttcgtgaactgttagttggggttgattatgtgtgggacggtgtaagggg
tttacctgtgtgttgtgttcagcatattaacaatagtgggggaggcttgggactttgtcc
ccattgcattaatgtaggggcttggtataatggatggaaatttcgagaatttaccccaga
tttggtgcggtgtagctgccatgtgggagcttctaatcccttttctgtgctaacctgcaa
aaaatgtgcttacctatctggattgcaaagctttgtagattatgagtaaagaaagtggca
aatggtgggaaagtgatgataaatttgctaaagctgtgtatcagcaatttgtggaatttt
atgaaaagcttactggaacagacttagagcttattcamatattaaaasatcattacaata
tttctttagataatcccctagaaaacccatcctctctgtttgacttagttgctcgtatta
aaaataaccttaaaaactctccagacttatatagtcatcattttcaaagtcatggacagt
tatctgaccacccccatgccttatcatccagtaacagtcatgcagaacctagaggagaaa
atgcagtattatctagtgaagacttacacaagcctgggcaagttagcgtacaactacccg
gtactaactatgttgggcctggcaatgagctacaagctgggcccccgcaaagtgctgttg
acagtgctgcaaggattcatgactttaggtatagccaactggctaagttgggaataaatc
catatactcattggactgtagcagatgaaga
>286565
aaactgggcaataaactacacttttgatttccctggaattaatgcagatgccctccaccc
agacctccaaaccaccccaattgtcacagacaccagtatcagcagcagtggtggtgaaag
ctctgaagaactcagtgaaagcagctttttttaacctcatcaccccaggcgcctggaacac
tgaaaccccgcgctctagtacgcccatccccgggaccagttcaggagaatcatctgtcgg
aagcccagtttcctccgaagttgtagctgcatcgtgggaagaagccttctacacaccttt
ggcagaccagtttcgtgaactgttagttggggttgattatgtatgggacggtgtaagggg
tttacctgtgtgttgtgtgcagcatattaacaatagtgggggaggcttgggactttgtcc
ccattgcattaatgtaggggcttggtataatggatggaaatttcgagaatttaccccaga
tttggtgcggtgtagctgccatgtgggagcttctaatcccttttctgtgctaacctgcaa
aaaatgtgcttacctgtctggattgcaaagctttgtagattatgagtaaagaaagtggca
aatggtgggaaagtgatgataaatttgctaaagctgtgtatcagcaatttgtggaatttt
atgagaagctgactggaacagacttagagcttattcaaatattaaaagatcattacaata

tttctttagataatcccctagaaaacccatcctctctgtttgacttagttgctcgtatta
aaaataaccttaaaaactctccagacttatatagtcatcattttcaaagtcatggacagt
tatctgaccacccccatgccttatcatccagtaacagtcatgcagaacctagaggagaaa
atgcagtattatctagtgaagacttacacaagcctgggcaagttagcgtacaactacccg
gtactaactatgttgggcctggcaatgagctacaagctgggcccccgcaaagtgctgttg
acagtgctgcaaggattcatgactttaggtatagccaactggctaagttgggaataaatc
catatactcattggactgtagcagatgaaga
>411433
aaactgggcaataaactacacttttgatttccctggaattaatgcagatgccctccaccc
agacctccaaaccaccccaattgtcacagacaccagtatcagcagcagtggtggtgaaag
ctctgaagaactcagtgaaagcagctttttttaacctcatcaccccaggcgcctggaacac
tgaaaccccgcgctctagtacgcccatccccgggaccagttcaggagaatcacttgtcgg
aagcccagtttcctccgaagttgtagctgcatcgtgggaagaagccttctacacaccttt
ggcagaccagtttcgtgaactgttagttggggttgattatgtgtgggacggtgtaagggg
tttacctgtgtgttgtgtgcagcatattaacaatagtgggggaggcttgggactttgtcc
ccattgcattaatgtaggggcttggtataatggatggaaatttcgagaattcaccccaga
tttggtgcggtgtagctgccatgtgggagcttctaatcccttttctgtgctaacctgcaa
aaaatgtgcttacctgtctggattgcaaagctttgtagattatgagtaaagaaagtggca
aatggtgggaaagtgatgataaatttgctaaagctgtgtatcagcaatttgtggaatttt
atgaaaagcttactggaacagacttagagcttattcaaatattaaaagatcattacaata
tttctttagataaccccctagaaaatccatcctctctgtttgacttagttgctcgtatta
aaaataaccttaaaaactctccagacttatatagtcatcattttcaaagtcatggacagt
tatctgaccacccccatgccttatcatccagtgacagtcatgcagaacctagaggagaag
atgcagtattatctagtgaagacttacacaagcctgggcaagttagcgtacaactacccg
gtactaactatgttgggcctggcaatgagctacaagctgggcccccgcaaagtgctgttg
acagtgctgcaaggattcatgactttaggtatagccaactggctaagttgggaataaatc
catatactcattggactgtagcagatgaaga
>414188
aaactgggcaataaactacacttttgatttccctggaattaatgcagatgccctccaccc
agacctccaaaccaccccaattgtcacagacaccagtatcagcagcagtggtggtgaaag
ctctgaagaactcagtgaaagcagctttttttaacctcatcaccccaggcgcctggaacac
tgaaaccccgcgctctagtacgcccatccccgggaccagttcaggagaatcacttgtcgg
aagcccagtttcctccgaagttgtagctgcatcgtgggaagaagccttctacacaccttt
ggcagaacagtttcgtgaactgttagttggggttgattatgtgtgggacggtgtaagggg
tttacctgtgtgttgtgtgcagcatattaacaatagtgggggaggcttgggactttgtcc
ccattgcattaatgtaggggcttggtataatggatggaaatttcgagaattcaccccaga
tttggtgcggtgtagctgccatgtgggagcttctaatcccttttctgtgctaacctgcaa
aaaatgtgcttacctgtctggattgcaaagctttgtagattatgagtaaagaaagtggca
aatggtgggaaagtgatgataaatttgctaaagctgtgtatcagcaatttgtggaatttt
atgaaaagcttactggaacagacttagagcttattcaaatattaaaagatcattacaata
tttctttagataaccccctagaaaatccatcctctctgtttgacttagttgctcgtatta
aaaataaccttaaaaactctccagacttatatagtcatcattttcaaagtcatggacagt
tatctgaccacccccatgccttatcatccagtgacagtcatgcagaacctagaggagaag
atgcagtattatctagtgaagacttacacaagcctgggcaagttagcgtacaactacccg
gtactaactatgttgggcctggcaatgagctacaagctgggcccccgcaaagtgctgttg
acagtgctgcaaggattcatgactttaggtatagccaactggctaagttgggaataaatc
catatactcattggactgtagcagatgaaga

>458100
aaactgggcaataaactacacttttgatttccctggaattaatgcagatgccctccaccc
agacctccaaaccactccaattgtcacagacaccagtatcagcagcagtggtggtgaaag
ctctgaagaactcagtgaaagcagctttttttaacctcatcaccccaggcgcctggaacac
tgaaaccccgcgctctagtacgcccatccccgggaccagttcaggagaatcatttgtcgg
aagcccagtttcctccgaagttgtagctgcatcgtgggaagaagccttctacacaccttt
ggcagaccagtttcgtgaactgttagttggggttgattatgtgtgggacggtgtaagggg
tttacctgtgtgttgtgtgcagcatattaacaatagtgggggaggcttgggactttgtcc
ccattgcattaatgtaggggcttggtataatggatggaaatttcgagaatttaccccaga
tttggtgcggtgtagctgccatgtgggagcttctaatcccttttctgtgctaacctgcaa
aaaatgtgcttacctgtctgggttgcaaagctttgtagattatgagtaaagaaagtggca
aatggtgggaaagtgatgataaatttgctaaagctgtgtatcagcaatttgtggaatttt
atgaaaagcttactggaacagacttagagcttattcaaatattaaaagatcattacaata
tttctttagataatcccctagaaaacccatcttctctgtttgacttagttgctcgtatta
aaaataaccttaaaaactctccagacttatatagtcatcattttcaaagtcatggacagt
tatctgaccacccccatgccttatcatccagtaacagtcatgcagaacctagaggagaaa
atgcagtattatctagtgaagacttacacaagcctgggcaagttagcgtacaactacccg
gtactaactatgttgggcctggcaatgagctacaagctgggcccccgcaaagtgctgttg
acagtgctgcaaggattcatgactttaggtatagccaactggctaagttgggaataaatc
catatactcattggactgtagcagatgaaga
>320189
aaactgggcaataaactacacttttgatttccctggaattaatgcagatgccctccaccc
agacctccaaaccaccccaattgtcacagacaccagtatcagcagcagtggtggtgaaag
ctctgaagaactcagtgaaagcagctttttttaacctcatcaccccaggcgcctggaacac
tgaaaccccgcgctctagtacgcccatccccgggaccagttcaggagaatcatttgtcgg
aagcccagtttcctccgaagttgtagctgcatcgtgggaagaagccttctacacaccttt
ggcagaccagtttcgtgaactgttagttggggttgattatgtgtgggacggtgtaagggg
tttacctgtgtgttgtgtgcagcatattaacaatagtgggggaggcttgggactttgtcc
ccattgtattaatgtaggggcttggtataatggatggaaatttcgagaatttaccccaga
tttggtgcggtgcagctgccatgtgggagcttctaatcccttttctgtgctaacttgcaa
aaaatgtgcttacctgtctggattgcaaagctttgtagattatgagtaaagaaagtggca
aatggtgggaaagtgatgataaatttgctaaagctgtgtatcagcaatttgtggaatttt
atgaaaagcttactggaacagatttagagcttattcaaatattaaaagatcattacaata
tttctttagataatcccctagaaaacccatcctctctgtttgacttagttgctcgtatta
aaaataaccttaaaaactctccagacttatatagtcatcattttcaaagtcatggacagt
tatctgaccacccccatgccttatcatccagtaacagtcatgcagaacctagaggagaaa
atgcagtattatctagtgaagacttacacaagcctgggcaagttagcgtacaactacccg
gtactaactatgttgggcctggcaatgagctacaagctgggcccccgcaaagtgctgttg
acagtgctgcaaggattcatgactttaggtatagccaactggctaagttgggaataaatc
catatactcattggactgtagcagatgaaga
>305713
aaactgggcaataaactacacttttgatttccctggaattaatgcagatgccctccaccc
agacctccaaaccaccccaattgtcccagacaccagtatcagcagcagtggtggtgaaag
ctctgaagaactcagtgaaagcagctttttttaacctcatcaccccaggcgcctggaacac
tgaaaccccgcgctctagtacgcccatccccgggaccagttcaggagaatcatctctcgg
aagcccagtttcctccgaagttgtagctgcatcgtgggaagaggccttctacacaccttt
ggcagaccagtttcgtgaactgttagttggggttgattatgtgtgggacggtgtaagggg

tttacctgtgtgttgtgtgcaacatattaacaatagtgggggaggcttgggactttgtcc
ccattgcattaatgtaggggcttggtataatggatggaaatttcgagaatttaccccaga
tttggtgcgatgtagctgccatgtgggagcttctaatcccttttctgtgctaacctgcaa
aaaatgtgcttacctgtctggattgcaaagctttgtagattatgagtaaagaaagtggca
aatggtgggaaagtgatgataaatttgctaaagctgtgtatcagcaatttgtggaatttt
atgaaaaggttactggaacagacttagagcttattcaaatattaaaagatcattataata
tttctttagataatcccctagaaaacccatcctctttgtttgacttagttgctcgcatta
aaaataaccttaaaaactctccagacttatatagtcatcattttcaaagtcatggacagt
tatctgaccacccccatgccttatcatccagtagcagtcatgcagaacctagaggagaag
atgcagtattatctagtgaagacttacacaagcctgggcaagttagcgtacaactacccg
gtactaactatgttgggcctggcaatgagctacaagctgggcccccgcaaagtgctgttg
acagtgctgcaaggattcatgactttaggtatagccaactggctaagttgggaataaatc
catatactcattggacggtagcagatgaaga
>743132
aaactgggcaataaactacacttttgatttccctggaattaatgcagatgccctccaccc
agacctccaaaccaccccaattgtcacagacaccagtatcagcagcagtggtggtgaaag
ctctgaagaactcagtgaaagcagctttttttaacctcatcaccccaggcgcctggaacac
tgaaaccccgcgctctagtacgcccatccccgggaccagttcaggagaatcatttgtcgg
aagcccagtttcctccgaagttgtagctgcatcgtgggaagaagccttctacacaccttt
ggcagaccagtttcgtgaactgttagttggggttgattatgtgtgggacggtgtaagggg
tttacctgtgtgttgtgtgcagcatattaacaatagtgggggaggcttgggactttgtcc
ccattgcattaatgtaggggcttggtataatggatggaaatttcgagaatttaccccaga
tttggtgcggtgtagctgccatgtgggagcttctaatcccttttctgtgctaacctgcaa
aaaatgtgcttacttgtctggattgcaaagctttgtagattatgagtaaagaaagtggca
aatggtgggaaagtgatgataaatttgctaaagctgtatatcagcaatttgtggaatttt
atgagaagcttactggaacagacttagagcttattcaaatattaaaagatcattacaata
tttctttagataatcccctagaaaacccatcttctctgtttgacttagttgctcgtatta
aaaataaccttaaaaactctccagacttatatagtcatcattttcaaagtcatggacagt
tatctgaccacccccatgccttatcatccagtaacagtcatgcagaacctagaggagaaa
atgcagtattatctagtgaagacttacacaagcctgggcaagttagcgtacaactacccg
gtactaactatgttgggcctggcaatgagctacaagctgggcccccgcaaagtgctgttg
acagtgctgcaaggattcatgactttaggtatagccaactggctaagttgggaataaatc
catatactcattggacggtagcagatgaaga
>553925
aaactgggcaataaactacacttttgatttccctggaattaatgcagatgccctccaccc
agacctccaaaccaccccaattgtcacagacaccagtatcagcagcagtggtggtgaaag
ctctgaagaactcagtgaaagcagctttttttaacctcatcaccccaggcgcctggaacac
tgaaaccccgcgctctagtacgcccatccccgggaccagttcaggagaatcacttgtcgg
aagcccagtttcctccgaagttgtaactgcatcgtgggaagaagccttctacacaccttt
ggcagaccagtttcgtgaactgttagttggggttgattatgtgtgggacggtgtaagggg
tttacctgtgtgttgtgtgcagcatattaacaatagtgggggaggcttgggactttgtcc
ccattgcattaatgtaggggcttggtataatggatggaaatttcgagaatttaccccaga
tttggtgcggtgtagctgccatgtgggagcttctaatcccttttctgtgctaacctgcaa
aaaatgtgcttacctgtctggattgcagagctttgtagattatgagtgaagaaagtggca
aatggtgggaaagtgatgataaatttgctaaagctgtgtatcagcaatttgtggaatttt
atgaaaagcttactggaacagacttagagcttatccaaatattaaaagaccattacaata
tttctttagataatcccctagaaaacccatcctctctgtttgacttagttgctcgtatta

aaaataaccttaaaaactctccagacttatatagtcatcattttcaaagtcatggacagt
tatctgaccacccccatgccttatcatccagtaacagtcatgcagaacctagaggagaag
atgcagtattatctagtgaagacttacacaagcctgggcaagttagcgtacaactacccg
gtactaactatgttgggcctggcaatgagctacaagctgggcccccgcaaagtgctgttg
acagtgctgcaaggattcatgactttaggtatagccaactggctaagttgggaataaatc
catatactcattggactgtagcagatgaaga
>559102
aaactgggcaataaactacacttttgatttccctggaattaatgcagatgccctccaccc
agacctccaaaccaccccaattgtcacagacaccagtatcagcagcagtggtggtgaaag
ctctgaagaactcagtgaaagcagctttttttaacctcatcaccccaggcgcctggaacac
tgaaaccccgcgctctagtacgcccatccccgggaccagttcaggagaatcacttgtcgg
aagcccagtttcctccgaagttgtaactgcatcgtgggaagaagccttctacacaccttt
ggcagaccagtttcgtgaactgttagttggggttgattatgtgtgggacggtgtaagggg
tttacctgtgtgttgtgtgcagcatattaacaatagtgggggaggcttgggactttgtcc
ccattgcattaatgtaggggcttggtataatggatggaaatttcgagaatttaccccaga
tttggtgcggtgtagctgccatgtgggagcttctaatcccttttctgtgctaacctgcaa
aaaatgtgcttacctgtctggattgcagagctttgtagattatgagtgaagaaagtggca
aatggtgggaaagtgatgataaatttgctaaagctgtgtatcagcaatttgtggaatttt
atgaaaagcttactggaacagacttagagcttatccaaatattaaaagaccattacaata
tttctttagataatcccctagaaaacccatcctctctgtttgacttagttgctcgtatta
aaaataaccttaaaaactctccagacttatatagtcatcattttcaaagtcatggacagt
tatctgaccacccccatgccttatcatccagtaacagtcatgcagaacctagaggagaag
atgcagtattatctagtgaagacttacacaagcctgggcaagttagcgtacaactacccg
gtactaactatgttgggcctggcaatgagctacaagctgggcccccgcaaagtgctgttg
acagtgctgcaaggattcatgactttaggtatagccaactggctaagttgggaataaatc
catatactcattggactgtagcagatgaaga
